# *In vitro* and *in vivo* effects of easily administered, low-toxic retinoid and phenylacetate compounds on human neuroblastoma cells

**DOI:** 10.1038/sj.bjc.6601108

**Published:** 2003-07-15

**Authors:** N Sidell, M Pasquali, S Malkapuram, A B Barua, T Wanichkul, R K Wada

**Affiliations:** 1 Division of Research, Department of Gynecology and Obstetrics, Emory University School of Medicine, 1639 Pierce Drive, Atlanta, GA 30322, USA; 2 Department of Pathology, University of Utah, Salt Lake City, UT 84108, USA; 3 Department of Biochemistry and Biophysics, Iowa State University, Ames, IA 50011, USA; 4 Cancer Research Center of Hawaii and the Kapiolani Health Research Institute, Honolulu, HI 96813, USA

**Keywords:** neuroblastoma, phenylacetate, retinoyl glucuronide, chemoprevention

## Abstract

We have investigated the effects of the low-toxic retinoid, all-*trans* retinoyl *β*-glucuronide (RAG) alone and in combination with the phenylacetate (PA) derivative 4-chloro-phenylacetate (4-CPA) on the human neuroblastoma cell line, LA-N-5. *In vitro* studies demonstrated that RAG and 4-CPA treatments alone showed differentiation-inducing activity on LA-N-5 cells, with 4-CPA found to be about three-fold more potent than the PA parent compound in inducing morphologic differentiation and growth inhibition. As previously reported for retinoic acid (RA) and PA, RAG and 4-CPA were significantly more effective in their antiproliferative effects on the cells than either agent alone. Pharmacologic studies of 4-CPA in mice demonstrated that blood plasma levels reached peak concentrations 4 h after bolus administration of the compound and showed slow clearance characteristics with an apparent half-life of 4–8 h. As opposed to PA, 4-CPA was found to be essentially odourless and readily consumed in drinking water, giving rise to steady-state blood plasma levels of 4-CPA in the near mM range. Continuous consumption of 4-CPA in this manner for up to 5 months demonstrated no apparent adverse effects on the mice. Long-term RAG- and/or 4-CPA-treatment of nude mice injected with LA-N-5 cells demonstrated that both compounds alone exhibit potent antitumour activity. Together, RAG plus 4-CPA was the most effective treatment for inhibiting established tumour growth. In contrast, 4-CPA alone was equally as effective as the combination for preventing tumour development. The potent *in vivo* antitumour effects of 4-CPA could not be accounted for by the known ability of PA compounds to induce expression of the RA nuclear receptor beta (RAR*β*) suppressor gene. Taken together, these findings demonstrate the possibility that RAG and/or 4-CPA may serve as effective, less-toxic alternatives to 13-cis RA, which is presently being utilised for nb therapy.

Neuroblastoma (nb) is a tumour of the sympathetic peripheral nervous system, originating in cells derived from the neural crest. It is the most common extracranial solid tumour in children, and comprises up to 50% of malignancies among infants (Gale *et al*, 1982). One characteristic of this disease is the long-recognised tendency of the tumours in a certain group of patients, designated clinically as stage 4S, to undergo spontaneous regression, at times accompanied by cellular differentiation (Evans *et al*, 1976). This property has prompted widespread interest in the use of retinoids as differentiation-inducing agents for the treatment of this disease. Matthay *et al* (1999) reported the results of a prospective double-blind trial in which 13-*cis* retinoic acid (13-*cis* RA) was given for 6 months to children with nb following consolidation therapy of their disease which included surgery, radiotherapy, chemotherapy, and/or bone-marrow transplantation. The results indicated that the 3-year event-free survival of patients taking 13-*cis* RA was almost double that of the control groups. These findings have now prompted the use of long-term administration of 13-*cis* RA in the setting of minimal residual disease as part of the standard therapy of nb. Thus, nb has become only the second cancer, after acute promyelocytic leukaemia (Piazza *et al*, 2001), to utilise retinoids as part of its paradigm for first-line therapy.

These encouraging clinical results have engendered a renewed interest in using retinoids in a safer and more effective manner for the treatment of nb. The present therapeutic protocol calls for six, 1-month cycles of 13-*cis* RA administration. Each cycle consists of 14 consecutive days of oral administration followed by 14 days of a drug-free ‘holiday’ period. The reasons for this drug-free period every 2 weeks include the severe hypervitaminosis A side effects as well as the induction of increased RA metabolism that can occur in children undergoing continuous treatment with 13-*cis* RA (Ruiz-Maldonado *et al*, 1998). Recently, we have demonstrated that long-term chronic dosing of mice with all-*trans* retinoyl *β*-glucuronide (RAG) is surprisingly lacking in toxicity and systemic adverse side effects that are usually associated with long-term retinoid therapy (Sidell *et al*, 2000). These studies showed that blood plasma concentrations of RAG in the micromolar ranges were maintained throughout the 2-month treatment protocol. Surprisingly, the data indicated that the plasma concentration of all-*trans* retinoic acid (RA) as a hydrolysis product of RAG, also did not decrease during the course of treatment, as opposed to decreased levels seen after chronic dosing with RA itself (Regazzi *et al*, 1997).

In studies aimed at finding ways to increase the efficacy of retinoid treatment of nb cells, we previously showed that the phenylalanine metabolite phenylacetate (PA) can both stimulate the differentiation of nb cells by itself and act synergistically with RA (Sidell *et al*, 1995). These experiments suggested that differentiation in the presence of both agents includes PA-induced effects that directly impact on the RA differentiation programme and require only relatively short priming periods, as well as RA-independent effects of PA which require the continued presence of this compound. One example of this former activity is illustrated by the finding that PA can enhance RA induction of nuclear retinoic acid receptor-*β* (RAR*β*) in nb cells, a molecular marker correlated with retinoid sensitivity and favourable prognosis (Cheung *et al*, 1998).

Taken together, the lack of adverse side effects seen with long-term RAG dosing along with the enhanced retinoid responses that have been demonstrated by nb cells when cotreated with PA, have prompted us to address the possibility that RAG/PA therapy might function as a more effective, nontoxic substitute for 13-*cis* RA in the treatment of nb. Our results demonstrate the biological activity of RAG on human nb cells, and suggest that the activity of this retinoid can safely be enhanced by *in vivo* combination treatment with the PA derivative 4-chlorophenylacetate (4-CPA).

## MATERIALS AND METHODS

### Chemicals and solvents

All-*trans*-retinoyl *β*-glucuronide was synthesised according to Becker *et al* (1996). The purity of RAG was determined by HPLC (Connor and Smit, 1987). Stock solutions of RAG were prepared by first dissolving it in dimethylsulphoxide to a concentration of 5 × 10^−2^ mM and then diluting it in complete medium to the indicated concentrations. 4-chloro-phenylacetate (4-CPA) was obtained from Sigma Chemical Co. (St Louis, MO, USA). Stock solutions were prepared by neutralising 4-chlorophenylacetic acid with NaOH and adjusting the volume with distilled water to give a final concentration of the sodium salt of 3 M. All other chemicals were purchased from Sigma unless otherwise indicated.

### Mice

Female athymic nude mice (5–6-week old) of a CD-1 heritage were obtained from Charles River (Wilmington, MA, USA). The mice were housed, maintained, and treated in accordance with NIH and UKCCCR guidelines for animal use and care under the supervision of Laboratory Animal Resources, Emory University (Workman *et al*, 1998). They received *ad libitum* a standard pellet diet (Purina, St Louis, MO, USA) and either tap water or during some treatment protocols tap water containing 4-CPA as described below. All food and water was sterilised by autoclaving before consumption.

### Cell culture and inoculation

The LA-N-5 human nb cell line was grown in HEPES-buffered RPMI 1640 medium supplemented with 10% heat-inactivated FBS, 50 IU ml^−1^ penicillin/streptomycin and 1 *μ*g amphotericin B (complete medium). The effects of various treatments on LA-N-5 cell proliferation was assessed by staining of cell protein with sulphorhodamine (SRB) as described (Han *et al*, 2001). Cells were prepared for inoculation by washing, resuspending in medium, counting with a haemocytometer and subcutaneous dorsal injection of approximately 10^7^ cells in a volume of 0.1 ml. Tumour growth was assessed weekly from the time when a nodule was first palpated. Tumour volumes were estimated according to the formula *V*=0.4*ab*^2^ with *a* and *b* being the perpendicular axes of the tumours and *b* being the smaller axis. Since there was some variation in tumour size when first palpated, growth was expressed as percent of initial volume.

### Acetylcholinesterase

Specific acetylcholinesterase (AChE) activity was measured as a biochemical index of the relative state of differentiation of treated and control LA-N-5 cells (Sidell *et al*, 1984). To measure the AChE activity, cells were grown in six-well tissue culture plates for 5 days in the absence or presence of the indicated concentrations of RAG and/or 4-CPA, washed twice with isotonic saline and harvested by vigorous shaking of the culture flask. After removal of saline, cells were frozen at −70°C, thawed by adding ice-cold 10 mM sodium phosphate buffer (pH 7.4) containing 0.5% Triton X-100 (1.5 ml 10^−6^ cells) and sonicated for 10 s. Acetylcholinesterase activity in samples of the homogenate was determined photometrically by following the hydrolysis of acetylthiocholine as previously described (Sidell *et al*, 1984). Protein concentrations were determined with a Sigma Bicinchoninic acid protein assay kit using BSA as the standard. Results, in nmol h^−1^ mg^−1^ protein, are expressed as means±s.e.m. of four independent experiments, each carried out in duplicate.

### RNA isolation, reverse transcriptase reaction, and real-time PCR

LAN-5 cells were treated with appropriate compounds for 2–3 days. Total RNA was then prepared by using TRIzol Reagent (GIBCO, BRL, Rockville, MD, USA). Reverse transcription was performed using the Perkin Elmer RT–PCR kit with 4 *μ*g RNA in a 20 *μ*l reaction volume according to the manufacturer's instructions. The reaction conditions were: 42°C for 1 h and 99°C for 5 min.

For real-time PCR, we used the Qiagen Master Mix kit and followed the vendor guidelines with some modifications. A total reaction volume of 25 *μ*l contained 12.5 *μ*l of Master Mix, 2 *μ*l of 25 mM MgCl_2_, and 0.25 *μ*l of 25 × SyBr Green (BioWhittaker Molecular Applications, Rockland, ME, USA). For RAR*β*, 5 *μ*l of cDNA was used and 3 *μ*l was used for GAPDH control. Samples were processed using the Cephied Smart Cycler software (Cephied Systems, Sunnyvale, CA, USA) under the following conditions: one denaturation cycle of 95°C for 30 s followed by 32 amplification cycles of 95°C for 10 s, 60°C for 15 s and 72°C for 40 s. Primers for amplifications were made by Sigma-Genosys and the sequences were as follows: GAPDH sense-5′-CCATGGAGAAGGCTGGG-3′; GAPDH antisense-5′-CAAAGTTGTCATGGATGAC-3′, (∼200 bp amplicon); RAR*β* sense-5′-CACTGGCTTGACCATCGC-3′; RAR*β* antisense-5′-GAGAGGTGGCATTGATCC-3′, (∼500 bp amplicon).

Melt curve analysis of each sample was supplemented with agarose gel electrophoresis of randomly selected samples to confirm the success of reactions. Fluorescence spectra were recorded during the annealing phase of the reaction. Second derivative analysis of the amplification curves was carried out to arrive at the threshold cycles for each sample. The following formula was used to arrive at the fold increase RAR*β* mRNA level for each sample:


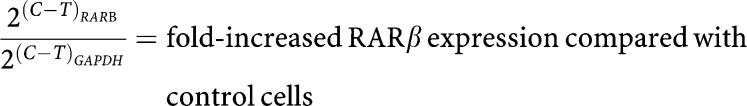


where *C* is the cycle threshold (CT) for RAR*β* or GAPDH mRNA detection in control samples, *T*=*CT* for RAR*β* or GAPDH mRNA detection in treatment samples.

### *In vivo* treatments

Groups of mice were treated with RAG and/or 4-CPA or solvent controls. RAG was administered by daily subcutaneous (s.c.) injections for the indicated time periods as previously described (Sidell *et al*, 2000). For treating with 4-CPA, the compound was given either by single intragastric administration (gavage) or admixed in their drinking water at the concentrations and time periods indicated in the long-term studies. At the indicated time after RAG and 4-CPA administration, mice were anaesthetised using Metofane (methoxyflurane, Mallinckrodt Veterinary, Mundelein, IL, USA). They were then exsanguinated until they expired by cardiac puncture into heparinised syringes. The blood was centrifuged for 12 min at 1200 **g** at room temperature and the plasma was recovered and stored at −20°C until analysis by gas chromatogrophy/mass spatrosopy (GC/MS). Plasma (500–600 *μ*l) was obtained from each mouse.

#### Extraction and analysis of 4-CPA from plasma samples

Quantitation of 4-CPA was performed by GC/MS following ethylacetate/ether extraction of plasma deproteinised with 7% perchloric acid, according to standard procedures (Thompson and Markey, 1975; Tanaka *et al*, 1980). Specifically, 250 *μ*l of plasma was admixed with 250 *μ*l of 7% perchloric acid. The mixture was centrifuged and the supernatant used for the analysis. The quantitation was accomplished by selective ion monitoring (SIM) using an external calibration curve obtained with a standard undergoing the same extracting procedure as the samples. Corrections for variations in the injections were made with an internal standard. The response was linear over a wide range (0–1700 nmol).

## RESULTS

### Morphologic differentiation

**Figure 1:**
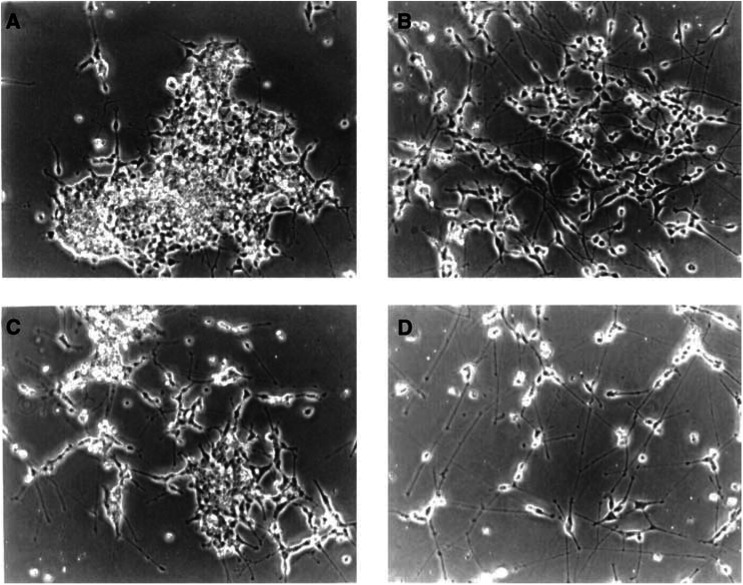
Morphology of LA-N-5 nb cells in the absence and presence of 4-CPA and/or RAG. Cultures were treated for 7 days with vehicle (**A**), 5 *μ*M RAG (**B**), 3 mM 4-CPA (**C**) or 3 mM 4-CPA+5 *μ*M RAG (**D**). Cells were plated at different densities (10^5^ to 5 × 10^5^ cells in 25 cm^2^ tissue culture flasks) to allow for the profound antiproliferative effect of combination treatment. Photographed under phase contrast (× 200).

We utilised the PA derivative 4-CPA in the present study because of its documented greater potency in terms of its differentiation-inducing activity in comparison with the parent compound (Pineau *et al*, 1996) and because of its ease in treating animals *in vivo* (see below). As shown in Figure 1

, 4-CPA induced neurite outgrowth from LA-N-5 cells. The temporal aspect of this effect was similar to that reported for PA (Sidell *et al*, 1995); it first became apparent after about 3 days of continuous exposure in culture. In contrast, while maximal increases in the formation of neurites occurred at PA concentrations between 5 and 10 mM, 4-CPA was found to be about three-fold more potent with maximal neurite-inducing activity occurring at about 3 mM.

As previously reported (Sidell *et al*, 2000), RAG also induced neurite sprouting from LA-N-5 cells, with maximal effects seen at RAG concentrations in the 5–10 *μ*M range after extended periods (>6 days). These morphologic changes are similar to those extensively reported after RA treatment of LA-N-5 cells (Sidell *et al*, 1983; Robson and Sidell, 1985), with the exception that maximal neurite outgrowth is seen with RA at somewhat lower concentrations (>1 *μ*M). In the presence of optimal concentrations of both agents, morphologic differentiation of LA-N-5 was noticeably enhanced; treatment with 3 mM 4-CPA plus 5 *μ*M RAG resulted in extensive neurite outgrowth which, after longer periods (approx. 2 weeks), formed an elaborate network of neurite bundles and thin fibres similar to that observed with combination RA and PA treatment (Sidell *et al*, 1995).

### Proliferation

**Figure 2:**
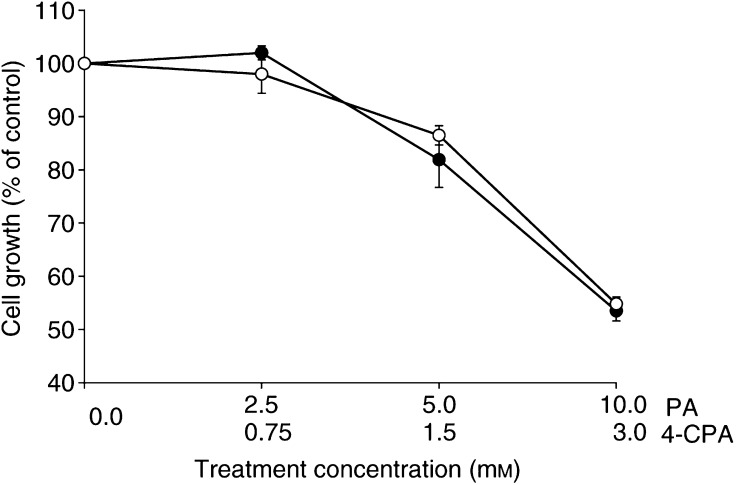
Dose–response curves showing antiproliferative effects of 4-CPA (*O*) in comparison to PA (•) on LA-N-5 cells as assessed by SRB protein staining after 5 days of treatment. Values are the averages (±s.e.m.) of at least three independent experiments for each treatment condition and are expressed as percent of control cultures as assessed by the SRB protein assay.

Previous studies have demonstrated the antiproliferative effects of RAG on LA-N-5 cells and indicated that this retinoid was 5–10-fold less potent than RA on a concentration-dependent basis (Sidell *et al*, 2000). Figure 2

shows the dose-dependent effects of 4-CPA on the growth of LA-N-5 cells as assessed by SRB protein staining. As seen, 50% growth inhibition was achieved at approximately 3 mM in these 5-day cultures, while little effect was found at 0.75 mM and below. For comparison, the growth inhibitory effects of PA were also tested in parallel cultures. The dose-dependent curves indicate that 4-CPA was about three-fold more potent than PA in inhibiting the growth of LA-N-5 cells. This greater potency of 4-CPA *vs* PA was consistent with our morphologic observations of the relative ability of the two compounds to induce neurite outgrowth.

**Figure 3:**
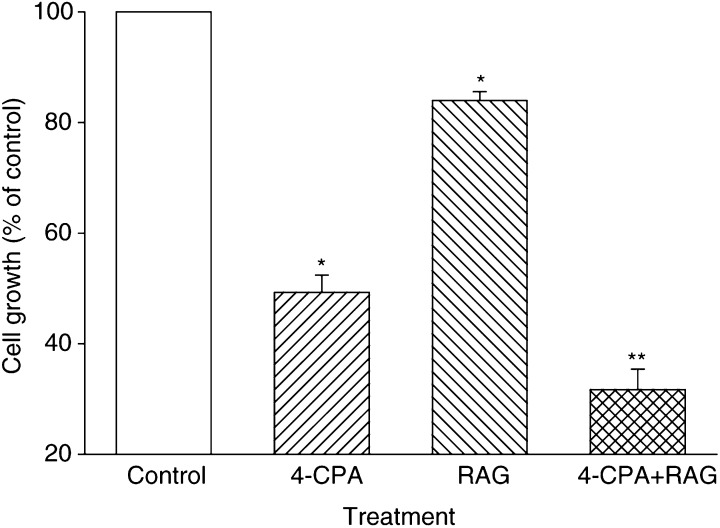
4-chloro-ph enylacetae and RAG are synergistic in inhibiting the growth of LA-N-5 cells. Cells were cultured in the presence of 4-CPA (3 mM), RAG (1 *μ*M), 4-CPA (3 mM)+RAG (1 *μ*M), or in the absence of added compounds as indicated. After 5 days, relative proliferation of the cultures was assessed by SRB protein staining. Columns represent the mean (±s.e.m.) of four independent experiments and are expressed as percent of control cultures as assessed by the SRB protein assay. ^*^ Indicates a significant difference between the single treatment as compared to the control. ^**^ Indicates significance between combination treatment and treatments with either 4-CPA or RAG alone. Student's *t*-test (two-tailed) was used for all statistical determinations, where a level of *P*<0.05 was considered statistically significant.

As previously reported for RA and PA, RAG and 4-CPA together were significantly more effective in their antiproliferative effects on LA-N-5 cells than one would expect from the combined action of two antiproliferative agents acting through independent mechanisms. For example, in Figure 3

, cell growth in the presence of 3 mM 4-CPA was around 50% of control, while that with 1 *μ*M RAG was 82%. Both agents together at these concentrations reduced cell growth to 30% of control, while 41% would be expected from the two agents acting independent of each other (0.82 × 0.50=41%).

### Acetylcholinesterase activity

**Figure 4:**
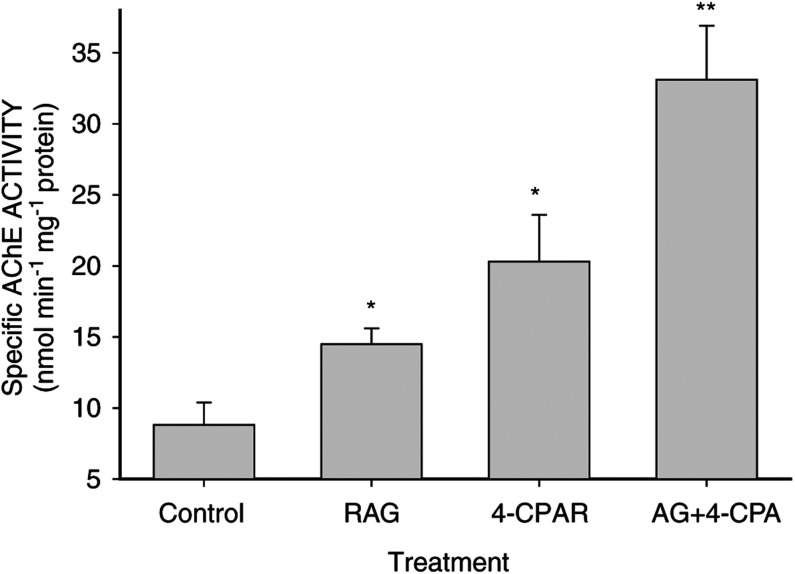
Effects of RAG and 4-CPA on AChE activity in LA-N-5 cells. Cells were cultured for 5 days in the presence of 5 *μ*M RAG, 3 mM 4-CPA, 5 *μ*M RAG+3 mM 4-CPA, or solvent control as indicated. Bars represent the mean±s.e.m. of four independent experiments. ^*^ Indicates a significant difference between the single treatment as compared to the control. ^**^ Indicates significance between combination treatment and treatments with either 4-CPA or RAG alone (*P*<0.05).

Concomitant with growth inhibition and neurite outgrowth, we have shown that AChE activity increases in LA-N-5 cells induced to differentiate with a variety of agents including RA and PA (Wuarin *et al*, 1991; Sidell *et al*, 1995). As a biochemical marker of LA-N-5 differentiation, we measured AChE activity in LA-N-5 cells treated with RAG and 4-CPA for 5 days. In preliminary experiments, we determined that AChE showed maximal increases when treated with either agent alone at RAG and 4-CPA concentrations of 5 *μ*M and 3 mM, respectively (data not shown). Previous work with RA and PA demonstrated maximal effects on AChE activity at concentrations of approximately 1 *μ*M and 5 mM, respectively (Sidell *et al*, 1995). Thus, the differences in the potency of 4-CPA *vs* PA and RAG *vs* RA to increase AChE was similar to that seen for their comparative effects on morphologic differentiation (neurite outgrowth) and growth of the cells. As seen in Figure 4

, there was a significant increase in specific AChE activity in cultures treated with a combination of 4-CPA and RAG compared with those treated with either agent alone. As previously reported, the induced increase in AChE activity generally paralleled the extent and complexity of neurite formation in the LA-N-5 cultures (Sidell *et al*, 1984, 1995; Wuarin *et al*, 1991).

### *In vivo* administration of 4-CPA

**Figure 5:**
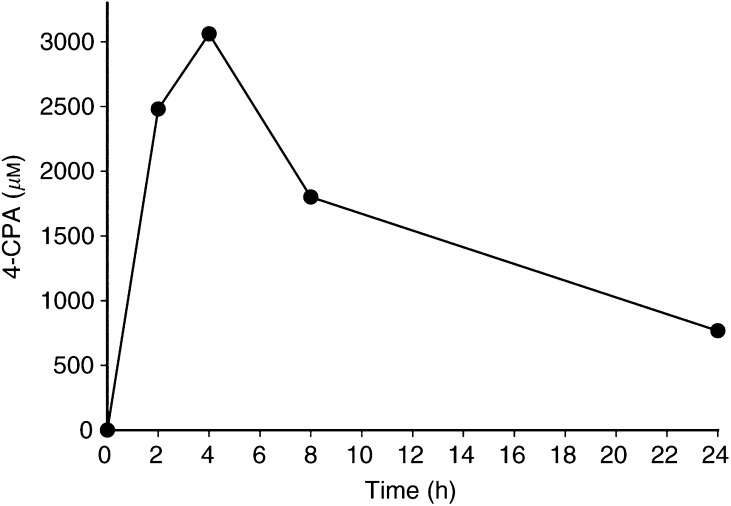
Plasma concentration of 4-CPA following a single gastric administration of 25.5 mg. Data represent the mean of two mice at each time point.

The mean concentrations of 4-CPA found in mouse plasma at various times during a 24 h-period after bolus administration of the compound (25.5 mg mouse^−1^) are shown in Figure 5

. We found the lower limit of detection of 4-CPA to be around 4 *μ*g ml^−1^ by the GC/MS procedure utilised. As shown, 4-CPA reached peak plasma concentrations 4 h after administration, achieving levels of 3.1 mM. 4-chloro-phenylacetate could not be detected in the plasma of any control (untreated) mouse. As previously determined with PA (Thibault *et al*, 1994), clearance of 4-CPA was relatively slow with an apparent half-life of 4–8 h. Even 24 h after dosing, plasma levels of 4-CPA>500 *μ*M was still detected.

As opposed to PA, 4-CPA is essentially odourless and readily consumed in drinking water. As such, admixing in drinking water provided a convenient method for chronic administration of 4-CPA for the long periods that may be required due to the long lag periods necessary for growth of LA-N-5 and other nb tumours in nude mice (Abemayor *et al*, 1990). Preliminary studies indicated that continuous consumption of up to 6 mg ml^−1^ 4-CPA in drinking water for up to 5 months showed no apparent adverse effects on the mice; there were no significant differences between 4-CPA-consuming and control groups in their water consumption, growth rate, social behaviour, or vitality. Higher doses of 4-CPA did show some toxicity in that the growth rate of the 4-CPA-consuming animals was reduced. Therefore, 6 mg ml^−1^ 4-CPA in drinking water was used in all our *in vivo* studies. Plasma was obtained by cardiac puncture from two groups of mice (3 mice group^−1^) that consumed either 6 (HD; high dose) or 3 mg ml^−1^ (LD; low dose) 4-CPA in their drinking water for 24 days. Analysis by GC/MS indicated that the mean (±s.e.m.) plasma concentration of 4-CPA in the HD and LD groups were (in *μ*M) 775 (±131) and 194 (±36), respectively. Plasma samples obtained from HD-treated mice at various other times up to 56 days of continuous 4-CPA consumption showed plasma levels of 4-CPA similar to those indicated above.

### Activation of RAR*β* as an indicator of LA-N-5 response by pharmacologic concentrations of compounds

**Figure 6:**
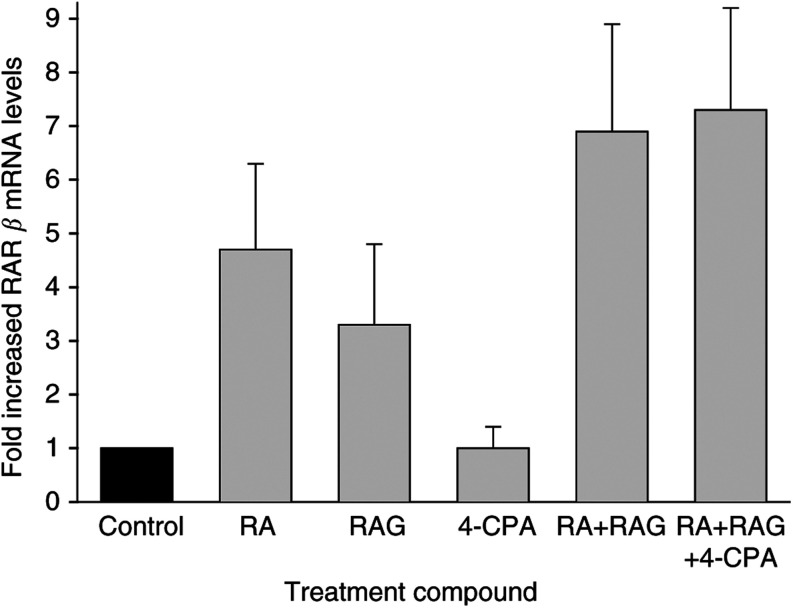
Effects of pharmacologic concentrations of 4-CPA and retinoids on RAR*β* expression. LA-N-5 cells were treated with RA (0.5 *μ*M), RAG (7.5 *μ*M), 4-CPA (0.75 mM), RA (0.5 *μ*M)+RAG (7.5 *μ*M), RA (0.5 *μ*M)+RAG (7.5 *μ*M)+4-CPA (0.75 mM), or solvent control as indicated for 2 days. Fold-increased RAR*β* mRNA levels were quantified by real-time RT–PCR as described in Materials and Methods. Columns represent the mean (±s.e.m.) of three experiments.

In human nb, as in many other tissues, RAR*β* shows low constitutive expression in untreated cells but is rapidly induced by retinoic acid (Wuarin *et al*, 1994). Previous studies by us (Sidell *et al*, 1998) and others (Cheung *et al*, 1998) have demonstrated that induction of RAR*β* plays a necessary role in the differentiation response of nb cells to retinoids. Furthermore, the data suggest that the magnitude of this induction can provide a molecular marker of the extent of the response. As such, we were interested in determining the effects on RAR*β* expression of retinoids and 4-CPA at concentrations measured in the mice blood plasma during our treatment. For these experiments, real-time RT–PCR was used to determine relative mRNA levels of RAR*β* in LA-N-5 cells treated for 2 days with RA, RAG, and 4-CPA at concentrations that were determined to be achieved in mouse blood plasma following chronic administration of RAG and 4-CPA as shown in our past (Sidell *et al*, 2000) and present work. To this end, our previous study showed that mean peak plasma levels of retinoids achieved during long-term daily administration of RAG by s.c. injection were approximately 0.5 *μ*M RA (as a hydrolysis product of RAG) and 7.5 *μ*M RAG. Thus, the treatment concentrations of RA, RAG, and 4-CPA utilised were 0.5 *μ*M, 7.5 *μ*M, and 0.75 mM, respectively. As can be seen in Figure 6

, RA or RAG alone induced a 3–5-fold increase in RAR*β* expression compared to controls. Together, these retinoids were essentially additive and caused about a seven-fold induction. 4-chloro-phenylacetate alone had no apparent effect on RAR*β* mRNA levels, and did not affect induction when combined with either RAG (date not shown) or when added along with both RAG and RA.

### Effects of *in vivo* treatments on LA-N-5 growth

**Figure 7:**
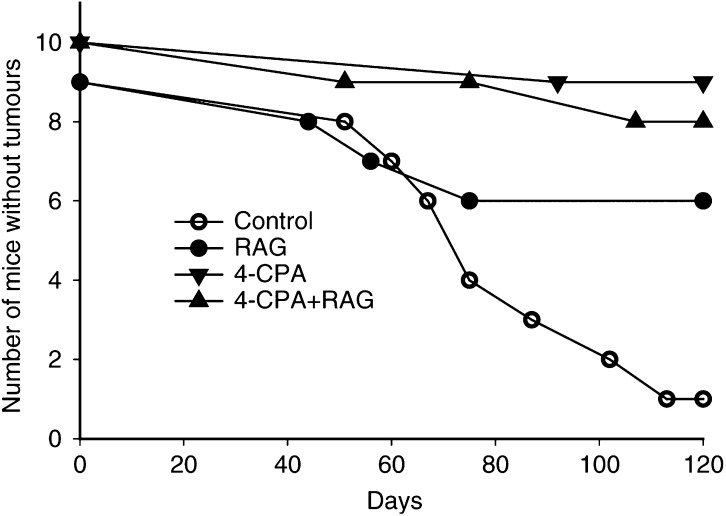
Development of tumours in *in vivo*-treated nude mice. Groups of animals containing nine or 10 mice were treated with 4-CPA (▾), RAG (•), 4-CPA+RAG (▴) or solvent controls (○) for 5 days before and 30 days after injection of 0.1 ml of 10^7^ LA-N-5 nb cells. Tumour development was recorded on the day that a nodule could first be definitively detected (∼3–5 mm diameter).

To determine the effects of treatments on tumour development, 4-CPA (6 mg ml^−1^ in drinking water) and/or RAG (25 *μ*mol kg^−1^ body weight) treatments were begun 3 days before tumour cell injections and then continued daily for 30 days after injection of nb cells (protocol A). Control animals were treated in a similar fashion except for the administration of solvents in place of RAG and/or 4-CPA. As previously seen with RAG (Sidell *et al*, 2000) or CPA alone (this report), no adverse effects were observed in any of the treatment groups. As seen in Figure 7

, in solvent-treated controls, 89% (eight out of nine) developed tumours by 120 days after injection. In contrast, only 33% (three out of nine) of the animals in the RAG-treated group developed tumours while 20% (two out of 10) and 10% (one out of 10) showed tumour development in the 4-CPA+RAG and 4-CPA groups, respectively. For tumours that did develop, there were no differences between the groups in terms of average tumour sizes (data not shown).

**Figure 8:**
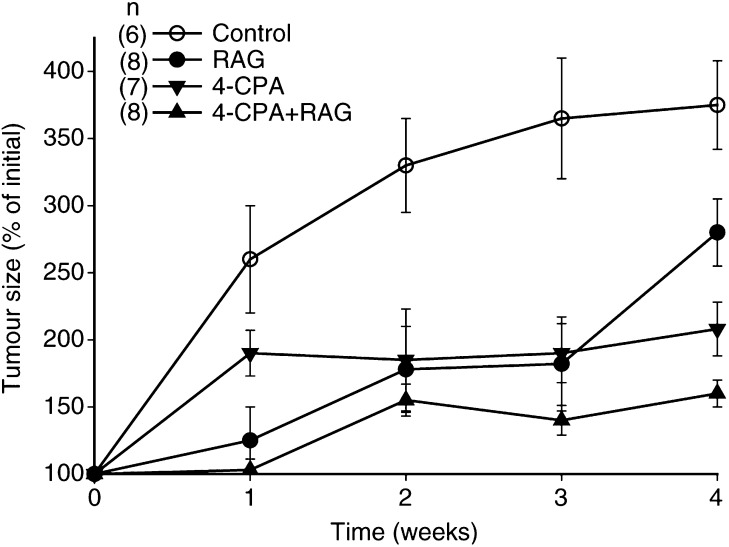
Growth of established tumours in *in vivo*-treated nude mice. Groups of animals were treated daily with either 4-CPA (▾), RAG (•), 4-CPA+RAG (▴), or solvent controls (○) for 28 days immediately following the development of palpable tumours. Tumour growth and volume were assessed weekly as described in Materials and Methods. Results represent mean (±s.e.m.) of six to eight animals/treatment group. On weeks 2 and 3, tumour size in all treatment groups was significantly reduced in comparison to that of controls only (*P*<0.05). On week 4, there was a significant difference in tumour size between any group of animals in comparison to any other treatment group (*P*<0.05).

To assess whether *in vivo* treatment could affect the growth of established tumours, groups of animals were treated for 28 days with solvent or 4-CPA/RAG as mentioned above immediately following the development of palpable tumours (3–6 mm diameters) (protocol B). During this time period, it was found that while tumours grew progressively in the control group, there was a diminished growth rate of tumours in animals in the treatment groups; from week 2 onwards, tumour growth in all the treatment groups was significantly reduced from that in the control group (Figure 8

). After the full 4-week treatment period, tumours in the 4-CPA+RAG group were significantly smaller than those in the other animals (*P*<0.05).

## DISCUSSION

The data presented in this report demonstrate the ability of an easily administered, nontoxic retinoid, RAG, to inhibit nb tumour development *in vivo*. In our previous work, we investigated both the pharmacokinetics of RAG during chronic s.c. administration and its conversion to RA (Sidell *et al*, 2000). We have chosen this route of administration for a number of reasons: (i) RAG was found to be poorly absorbed from the gastrointestinal track following oral dosing (Barua, 1997); (ii) elimination of RAG following s.c. injection has been shown to be relatively slow, showing a half-life of greater than 10 h (Nau *et al*, 1996; Sidell *et al*, 2000); (iii) s.c. injection would be a simple, nontraumatic means of chronic daily treatment of infants with nb as well as a convenient means of long-term dosing in animal models (*vs* oral or intravenous routes). Previous results indicated that the peak (2 h) plasma levels of both RAG and its hydolysis product RA were similar at all times throughout the entire dosing period (up to 2 months) and showed RAG and RA plasma levels of around 7.0 and 0.5 *μ*M, respectively. Thus, in contrast to what occurs during administration of RA itself (Regazzi *et al*, 1997), it appeared that the production of RA from the continuous hydrolysis of persistent RAG levels did not result in the induction of cytochrome *P*450 oxidative enzymes. Finally, the studies demonstrated that retinol levels did not change significantly during the course of RAG treatment as opposed to that reported with the long-term administration of some other retinoids such as RA and 13-*cis* RA (Barua *et al*, 1997; Keilson *et al*, 1979).

Since PA can markedly enhance the differentiation-inducing effects of retinoids on nb cells *in vitro*, we were interested in determining if the antitumour activity of this combination of agents also extended to nb growth and/or tumour development *in vivo*. For these studies, we utilised the PA derivative 4-CPA. It was previously reported that halogen substitution on the aromatic ring of PA *para* to the alkylcarboxyl group can increase the potency by several fold in terms of its antiproliferative activity on several types of tumours (Hudgins *et al*, 1995). Among the derivatives found to be more potent than the parent compound, 4-CPA inhibited the growth of prostate carcinoma, glioblastoma, and melanoma with a potency 3–4 times that of PA. Our *in vitro* data demonstrated that this enhanced activity of 4-CPA over that of PA extends to nb cells. Moreover, we have found that 4-CPA lacks the adverse odour often associated with PA, and is therefore much more easily administered to mice orally admixed with drinking water. As such, we utilised this convenient method for chronic adminstration of 4-CPA to mice for relatively long periods of time. In initial experiments to explore the feasibility of using this method for achieving therapeutic blood levels *in vivo*, we determined that continuous consumption of up to 6 mg ml^−1^ 4-CPA in drinking water for 5 months showed no apparent adverse effects on the mice. Longer time periods were not tested. Using this methodology, steady-state plasma levels of over 750 *μ*M 4-CPA were achieved. This concentration compares favourably with plasma concentrations of 500–1000 *μ*M PA that can be achieved in humans and rodents by administering either PA itself or its prodrug phenylbutyrate (PB) by a variety of procedures (e.g., continuous infusion, injection, oral) (Thibault *et al*, 1994; Jung, 2001). As with 4-CPA, these concentrations of PA or PB have shown no adverse side effects in mice (Prasanna *et al*, 1995). In humans, the only side effects reported during therapeutic treatments with PA derivatives have been nausea and somnolence (Batshaw and Monahan, 1987).

In our studies, there were no significant differences in weight gain, water consumption, social behaviour, or apparent vitality between any of the treatment and control groups. In the groups where RAG was part of the treatment paradigm, there were no observable clinical manifestations of retinoid toxicity such as alopecia and scaly skin (Teelmann *et al*, 1993). Since our previous (Sidell *et al*, 2000) and present pharmacokinetic studies of RAG and 4-CPA indicated that biologically active blood levels of RAG, RA, and 4-CPA can be maintained during chronic long-term administration of the compounds, we assessed whether such levels could have therapeutic significance in terms of reduced *in vivo* tumour growth. For these experiments, two *in vivo* treatment protocols were utilised; the first where treatment was begun 3 days before tumour injection and continued for 30 days after injection (protocol A), and the second where treatment was administered for 28 days starting immediately after the appearance of palpable tumours (protocol B). As such, protocol A was designed as a model to evaluate the ability to affect the clinical remission time and subsequent regrowth of metastatic lesions in patients following consolidation therapy such as the setting in which 13-*cis* RA is now used. On the other hand, protocol B may be considered a model for testing the potential efficacy on established, primary nb tumour growth. Previous trials of 13-*cis* RA as well as other retinoids on progressive tumour growth in nb patients have demonstrated little, if any, efficacy (Reynolds and Lemons, 2001).

Our data showed that the proliferative capacity of the LA-N-5 nb cells was inhibited by all the *in vivo* treatments compared to controls. Of the two protocols used, protocol A in which treatment was started before tumour cell injection appeared to be the most effective in increasing the proportion of tumour-free animals. These results suggest that the treatments may be very effective in the setting of minimal residual disease or prevention of metastasis. Recent developments of metastatic mouse models of nb involving orthotopic implantation of tumour cells (Moats *et al*, 2000; Khanna *et al*, 2002) should provide a powerful tool for testing this hypothesis. In the presence of established tumours (protocol B), the treatments also inhibited tumour growth, although we could not demonstrate a concomitant increase in morphologic or biochemical differentiation of the nb cells (data not shown). These findings indicated that RAG alone was at least as effective as that previouly demonstrated with RA in an identical *in vivo* nude mouse model (Abemayor *et al*, 1990). Although combination RAG plus 4-CPA was shown to be the most effective treatment for inhibiting established tumour growth, this combination treatment was not more effective for preventing tumour development. Thus, only one out of 10 mice developed a tumour when being treated with 4-CPA alone. Although 4-CPA has previously not been tested *in vivo*, treatment of mice with only its parent compound PA in conjunction with MCF-7 breast cancer cell inoculation was likewise shown to be remarkedly effective in preventing tumour development (Di Benedetto *et al*, 2002).

We have demonstrated that PA can enhance RA induction of RAR*β* in human nb cells, an event which has been implicated as being necessary for RA-mediated growth inhibiton and differentiation of this cell type (Sidell *et al*, 1998). These *in vitro* findings were supported by Cheung *et al* (1998), who found strong correlations between favourable nb patient prognosis and RAR*β* expression, and that high-level expression of this receptor can profoundly inhibit nb cell growth even in the absence of exogenous retinoid. Therefore, we were interested in determining whether the inhibitory effects of the different treatments correlated with RAR*β* expression at retinoid and 4-CPA concentrations that were achieved *in vivo*. Quantitation of RAR*β* mRNA levels by real-time PCR suggests that this was not the case. Indeed, 4-CPA alone was found to be a potent inhibitor of tumour development and growth, but did not enhance RAR*β* expression compared to controls at 4-CPA blood concentrations that were achieved *in vivo*. Furthermore, treatment of LA-N-5 cells with concentrations of 4-CPA that showed maximal growth inhibitory effects *in vitro* (3 mM), only modestly (<two-fold) enhanced RAR*β* mRNA levels as compared to controls (data not shown). A similar small increase of RAR*β* mRNA levels was also seen following treatment with concentrations of PA that showed marked antiproliferative activity (Sidell *et al*, 1995, 1998). Thus, although it is possible that plasma concentrations of 4-CPA did not accurately reflect tumour tissue levels of the compound, it is clear that its potent antitumour effects are not mediated through enhanced RAR*β* signalling. Previous *in vitro* findings have revealed retinoid-independent effects of PA derivatives such as inhibition of protein prenylation, histone deacetylation activity, and activation of peroxisome-proliferator activated receptors (Hudgins *et al*, 1995; Pineau *et al*, 1996; Jung, 2001). Whether these activities play a role in the *in vivo* antitumour activity of 4-CPA is presently unknown. In light of the recent results demonstrating the effectiveness of retinoids in preventing nb recurrences in children (Matthay *et al*, 1999), our results offer encouragement that RAG and/or PA compounds may serve as effective, less-toxic alternatives to 13-*cis* RA therapy, which is now being utilised in the clinic.
